# Potential of pomegranate fruit extract (*Punica granatum* Linn.) to increase vascular endothelial growth factor and platelet-derived growth factor expressions on the post-tooth extraction wound of *Cavia cobaya*

**DOI:** 10.14202/vetworld.2017.999-1003

**Published:** 2017-08-27

**Authors:** Intan Nirwana, Priyawan Rachmadi, Devi Rianti

**Affiliations:** Department of Dental Material, Faculty of Dental Medicine, Universitas Airlangga, Jl. Mayjen Prof. Dr. Moestopo No 47 Surabaya, Jawa Timur, 60132, Indonesia

**Keywords:** platelet-derived growth factor, *Punica granatum* Linn. fruit, tooth extraction wound, vascular endothelial growth factor

## Abstract

**Background:**

Pomegranates fruit extracts have several activities, among others, anti-inflammatory, antibacterial, and antioxidants that have the main content punicalagin and ellagic acid. Pomegranate has the ability of various therapies through different mechanisms. Vascular endothelial growth factor (VEGF) function was to form new blood vessels produced by various cells one of them was macrophages. Platelet-derived growth factor (PDGF) was a growth factor proven chemotactic, increased fibroblast proliferation and collagen matrix production. In addition, VEGF and PDGF synergize in their ability to vascularize tissues. The PDGF function was to stabilize and regulate maturation of new blood vessels. Activities of pomegranate fruit extract were observed by measuring the increased of VEGF and PDGF expression as a marker of wound healing process.

**Aim:**

To investigate the potential of pomegranate extracts on the tooth extraction wound to increase the expression of VEGF and PDGF on the 4^th^ day of wound healing process.

**Materials and Methods:**

This study used 12 *Cavia cobaya*, which were divided into two groups, namely, the provision of 3% sodium carboxymethyl cellulose and pomegranate extract. The 12 *C. cobaya* would be executed on the 4^th^ day, the lower jaw of experimental animals was taken, decalcified about 30 days. The expression of VEGF and PDGF was examined using immunohistochemical techniques. The differences of VEGF and PDGF expression were evaluated statistically using t-test.

**Results:**

Statistically analysis showed that there were significant differences between control and treatment groups (p<0.05).

**Conclusion:**

Pomegranate fruit extract administration increased VEGF and PDGF expression on post-tooth extraction wound.

## Introduction

Tooth extraction is a surgery involving bone tissue and soft tissues of the oral cavity. Tooth extraction is also considered as a curative effort most frequently performed as described in the health profiles of East Java in 2014 showing that 201,922 of 305,400 basic dental services delivered in health centers were related to tooth extraction [1]. Unfortunately, tooth extraction can cause complications, such as bleeding, infection, fracture, and dry socket [2]. Impaired wound healing after the tooth extraction even can also become unfavorable for the next prosthodontic treatment.

Therefore, acceleration of wound healing process after tooth extraction is very necessary. Periodontal tissue destruction due to tooth extraction is an inflammation [3]. This condition can make macrophages activated, and can also trigger synthesis of both cytokines that have pro-inflammatory activities, such as interleukin (IL)-1, IL-6, IL-8, and tumor necrosis factor α [4], as well as another cytokine, namely, IL-10 serving as regulator [5]. In addition to cytokines growth factor plays an important role in the wound healing process [6].

Inflammation actually is a normal response to injury, but it must be controlled since it can cause negative effects on surrounding tissues [7]. Inflammation usually occurs in response to trauma, chemical irritation, and infection caused by bacteria or viruses. The healing process occurs in three main stages, namely, hemostatic and inflammatory stage, proliferation stage, and remodeling stage. Although granulation occurred at the stage of proliferation, angiogenesis begins immediately after the injury and is mediated through the wound healing process as a whole [8].

Increased angiogenic growth factor, plays an important role in the wound healing process, such as vascular endothelial growth factor (VEGF) and platelet-derived growth factor (PDGF). VEGF is a signaling protein triggering the growth of new blood vessels during the healing process. In other words, the protein will stimulate new blood vessel growth after injury. VEGF is produced by many cells one of which is macrophage. VEGF also plays an important role in bone and blood formations, necessary for the wound healing process [9].

In normal wound healing process, formation of granulation tissue containing fibrovascular composed of fibroblast, collagen, and blood vessels as markers of the healing response is important. Those vascular components depend on angiogenesis, in which new blood vessels appear on the 3^rd^ day after the injury (wounding). Therefore, this research observed increased expression of VEGF.

PDGF, on the other hand, is the first chemotactic growth factor for migration of cells to the injured area, such as neutrophils, monocytes, and fibroblasts. PDGF can increase fibroblast proliferation and extracellular matrix production due to those cells. PDGF can also stimulate fibroblasts to produce collagen matrix and to induce myofibroblast phenotype in those cells; therefore, PDGF has a major role in wound healing [10].

Furthermore, pomegranate has been used traditionally as medicine [11,12]. Pomegranate has various therapeutic abilities through different mechanisms such as anti-inflammatory, antibacterial, and antioxidants [12,13]. The main compound contained in pomegranates is polyphenols composed of punicalagin and ellagic acid (EA). EA has anti-inflammatory activity, degrading IL-6 by inhibiting nuclear factor-kappa B (NF-kB) [14].

For those reasons, it is necessary to conduct a research on the use of pomegranate extract on tooth extraction wound to accelerate the healing process by observing the increased expressions of VEGF and PDGF. Thus, this research aimed to investigate the potential of pomegranate extract (*Punica granatum* Linn.) to increase the expressions of VEGF and PDGF in the post-tooth extraction wound.

## Materials and Methods

### Ethical approval

This research was approved by the Ethical Committee of the Faculty of Dental Medicine, Universitas Airlangga, Indonesia.

### Experimental design

This research was a laboratory experimental research with a post-test only control group design. *C. cobaya* used in this research were 2-3 month old and weighed 250-350 g. 12 *C. cobaya* were adapted for 1 week before the treatment, and then randomly divided into two groups, namely, control group and treatment group. Each of the group consisted of six *C. cobaya*. During the study, all animals were given with standard chow and tap water *ad libitum*.

### Research material

Materials used in this research were pomegranate extract with standardized to 40% EA using high-performance liquid chromatography methods (Xi’an Biof Bio-Technology Co., Ltd. China.), sodium carboxyl methyl cellulose (CMC-Na), ketamine HCl and diazepam, non-absorbable thread, 10% ethylene diamine tetra acetic acid (EDTA), VEGF monoclonal antibody (Abcam), and PDGF monoclonal antibody (Sigma).

### Research method

The standardized pomegranate extracts used in this research was dissolved in 3% CMC-Na. 3% CMC-Na gel was made by dissolving 3 g of CMC-Na in 100 ml of warm water slowly to obtain a homogeneous solution. 2.5% pomegranate extract gel was made of 2.5 g of pomegranate extract standardized to 40% EA and dissolved in 97.5 g of 3% CMC-Na gel. Meanwhile, 3% CMC-Na gel was applied to the post-tooth extraction sockets of those *C. cobaya* in the control group. The treatment group was given with the standardized pomegranate extracts at a concentration of 2.5%.

The combination of ketamine HCl and diazepam was used to anesthetize those animals (1:1 cc, with a dose of 1 ml/kg b.m. intramuscularly) [15]. Leftover food was cleaned from their left mandibular incisor with water spray using a syringe and dried. Sterile elevator was used to separate the right and left incisors of those *C. cobaya* and their left mandibular incisor was extracted using sterile needle holder with the direction of movement toward the lingual aspect. Pomegranate extract gel was applied into the tooth sockets of those animals in the treatment group using insulin syringe with a dose of 0.1 ml per socket. The extraction wound in both groups then was sutured using non-absorbable thread. The *C. cobaya* in both the control and treatment groups was sacrificed on day four, and then their mandible was taken. The mandible of those animals was immersed in buffered formalin for 24 h. Buffered formalin then was replaced with 10% EDTA to decalcification for approximately 30 days until their mandibular bone tissue, and their teeth became soft and could be cut.

The tissue blocks were removed and embedded in paraffin, furthermore, longitudinal serial cross section of 5 µm tissue blocks were placed on poly-L-lysine coated slides for the purpose of performing immunohistochemistry. The slide sections were immersed in target retrieval solution (DAKO) and heated in microwave oven at 98°C for 20 min (maximum power 700 W) and then cooled at room temperature; anti-VEGF (Abcam) and anti-PDGF (Sigma) primary antibodies were used. The sections were incubated with each primary antibody as mentioned above for 1 h, after rinsing thrice in DAKO wash buffer (Tris-buffered saline [TBS]), the sections were then incubated with biotinylated secondary antibody using the kit (LSAB system 2-HRP) for 1 hour at room temperature. Following TBS rinses, the sections were incubated with streptavidin-horseradish peroxidase conjugate for additional 30 minutes at room temperature (DAKO), followed by a course of incubation diaminobenzidine (DAB) DAKO [16].

The expressions of VEGF and PDGF were measured by calculating the number of cells (macrophage and fibroblast) expressing VEGF and PDGF. The total number of immunoreactive cells was tabulated as data and then analyzed using a t-test to determine differences in the expressions of VEGF and PDGF.

## Results

**Figure-1:**
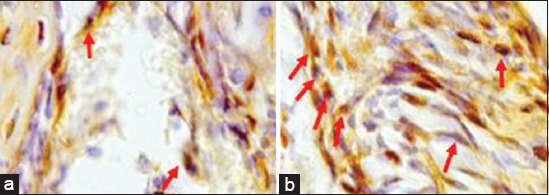
Vascular endothelial growth factor expressions in the control group (a) and in the treatment group (b).

**Figure-2:**
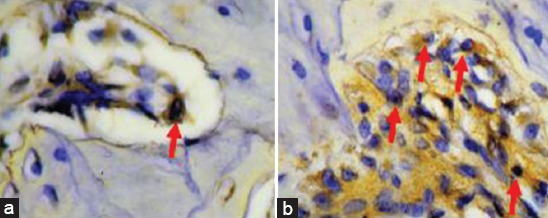
Platelet-derived growth factor expressions in the control group (a) and in the treatment group (b).

The number of VEGF expressed by macrophages and fibroblasts in the control group was less than in the treatment group (Figure-1). Similarly, the number of PDGF expressed by macrophages in the control group was less than in the treatment group (Figure-2). Results of the analysis of VEGF and PDGF expressions in both the control and treatment groups using the t-test also showed that there was a significant difference in VEGF and PDGF expressions in both groups (p<0.005). The mean and standard deviations of VEGF and PDGF in both the control and treatment groups were 14.50±2.258; 22.33±3.077; 12.50±2.588; and 22.67±3.141, respectively (Table 1).

**Table-1:** Mean and standard deviations of VEGF and PDGF expressions in the control group and the treatment group.

Group	N	VEGF			PDGF		
	
		Mean±SD	Value t	Sig	Mean±SD	Value t	Sig
C	6	14.50±2.258	5.027	0.001	12.50±2.588	6.118	0.000
T	6	22.33±3.077			22.67±3.141		

VEGF=Vascular endothelial growth factor, PDGF=Platelet-derived growth factor, SD=Standard deviation

## Discussion

Wound after tooth extraction can provide a suitable environment for the growth of microorganisms. This condition then contributes to impede or prolong the healing process. During inflammation phase, cells (neutrophils, macrophages, fibroblasts, and endothelial cells) produce reactive oxygen species (ROS), thereby causing a signal inflammation and protecting the wound from the invasion of microorganisms. Excessive ROS, however, will activate IkB kinase and IkB that will be phosphorylated, resulting in degradation of IKB and NF-kB protein will be released. NF-kB will translocate into the nucleus and drive target gene expression such as inflammatory cytokines [17]. ROS can decrease the rate of wound healing and cause damage to the surrounding cells.

Pomegranate extract can decrease IKK activation, and IKB degradation can be inhibited after tooth extraction. This condition then will lead to a decrease in NF-kB translocation. The decreased NF-kB translocation is accompanied by the decrease production of pro-inflammatory cytokine IL-6 [18]. It proves that pomegranate extract that contained EA and punicalagin has anti-inflammatory activity. Consequently, the inflammatory phase of the wound healing process can be regulated properly.

Angiogenesis, the growth of new blood vessels from existing blood vessels, is an important aspect of the healing process. The improvement of blood flow to the damaged tissue can supply oxygen and nutrients needed to support the growth and function of reparative cells. Therefore, the success of the wound healing depends on angiogenesis, the growth of new capillaries of blood vessel. The new capillaries usually will appear first in the wound 3-5 days after the injury [19].

Results of this research showed that there was a significant increase in VEGF expression on the 4^th^ day in the treatment group with the provision of pomegranate extract compared to the control group. The increased VEGF is very essential in angiogenesis during the wound healing process since it can produce vigorous angiogenic response. Thus, it can be said that pomegranate extract has pro-healing effects on injured area.

The 4^th^ day after administration of pomegranate extract, there were many macrophages in the injured area. Macrophages secrete VEGF, which stimulates the endothelial cells to migrate into the clot area, to proliferate, and to form new blood vessels [10]. These findings are consistent with a research conducted by Johnson and Wilgus [20] that explain VEGF expression will increase on days 3-5 and will decrease from day 7 to 14 after injury. These conditions indicate a normal healing process. Results of some previous researchers even reveal that there is a positive correlation between VEGF level and VEGF activity and the amount of granulation tissue formed in healthy rat [21]. Different conditions occur in diabetic animal, where the levels and activity of the VEGF decreases and followed by a decrease in the formation of granulation tissue [22,23].

VEGF is also considered as a major angiogenic agent stimulating migration, proliferation, and differentiation of endothelial cells. VEGF, thus, is known as a strong positive regulator of angiogenesis and endothelial cell functions needed for new blood vessel formation, such as proliferation, migration, differentiation, and survival [24].

In this research, the increased VEGF expression was accompanied with the increased PDGF expression. VEGF and PDGF synergize in their ability to vascularize tissues [25]. PDGF appears to transduce its signal through macrophages, and may also trigger the activation of feedback loops and the synthesis of both endogenous PDGF as well as other growth factors, thereby enhancing the cascade of tissue repair processes required for a fully healed wound. Newly forming blood vessels must be stabilized or matured under influenced of PDGF [26].

In the normal wound healing process, the expression of PDGF is a very important factor since its deficiency leads to abnormal and poorly-formed immature blood vessels [8,27]. Similarly, previous research on diabetic experimental animals shows that decreased PDGF expression can result in delayed wound healing process.

## Conclusion

The provision of pomegranate extract can increase VEGF and PDGF expression, leading to the acceleration of the healing process.

## Authors’ Contributions

IN has designed the plan of research work and a research coordinator. PR and DR carried out the laboratory work and analyzed the results. IN and DR drafted and revised the manuscript. All authors read and approved the final manuscript.
